# Effectiveness of ChAdOx1 vaccine in older adults during SARS-CoV-2 Gamma variant circulation in São Paulo

**DOI:** 10.1038/s41467-021-26459-6

**Published:** 2021-10-28

**Authors:** Matt D. T. Hitchings, Otavio T. Ranzani, Murilo Dorion, Tatiana Lang D’Agostini, Regiane Cardoso de Paula, Olivia Ferreira Pereira de Paula, Edlaine Faria de Moura Villela, Mario Sergio Scaramuzzini Torres, Silvano Barbosa de Oliveira, Wade Schulz, Maria Almiron, Rodrigo Said, Roberto Dias de Oliveira, Patricia Vieira Silva, Wildo Navegantes de Araújo, Jean Carlo Gorinchteyn, Jason R. Andrews, Derek A. T. Cummings, Albert I. Ko, Julio Croda

**Affiliations:** 1 Department of Biostatistics, College of Public Health & Health Professions, University of Florida, Gainesville, FL USA; 2 Barcelona Institute for Global Health, ISGlobal, Barcelona, Spain; 3 Pulmonary Division, Heart Institute (InCor), Hospital das Clinicas HCFMUSP, Faculdade de Medicina, Universidade de São Paulo, São Paulo, São Paulo Brazil; 4 Department of Epidemiology of Microbial Diseases, Yale School of Public Health, New Haven, CT USA; 5 Disease Control Coordination of the São Paulo State Department of Health, São Paulo, São Paulo Brazil; 6 Municipal Health Secretary of Manaus, Manaus, Amazonas Brazil; 7 Pan American Health Organization, Brasília, Distrito Federal Brazil; 8 Universidade de Brasília, Brasília, Distrito Federal Brazil; 9 Department of Laboratory Medicine, Yale University School of Medicine, New Haven, CT USA; 10 State University of Mato Grosso do Sul - UEMS, Dourados, Mato Grosso do Sul Brazil; 11 Universidade Federal de Mato Grosso do Sul - UFMS, Campo Grande, Mato Grosso do Sul Brazil; 12 National Institute for Science and Technology for Health Technology Assessment, Porto Alegre, Rio Grande do Sul Brazil; 13 Health Secretariat of the State of São Paulo, São Paulo, São Paulo Brazil; 14 Division of Infectious Diseases and Geographic Medicine, Stanford University, Stanford, CA USA; 15 Department of Biology, University of Florida, Gainesville, FL USA; 16 Emerging Pathogens Institute, University of Florida, Gainesville, FL USA; 17 Instituto Gonçalo Moniz, Fundação Oswaldo Cruz, Salvador, Bahia Brazil; 18 Fiocruz Mato Grosso do Sul, Fundação Oswaldo Cruz, Campo Grande, Mato Grosso do Sul Brazil

**Keywords:** Viral infection, Epidemiology

## Abstract

A two-dose regimen of the Oxford-AstraZeneca (ChAdOx1) Covid-19 vaccine with an inter-dose interval of three months has been implemented in many countries with restricted vaccine supply. However, there is limited evidence for the effectiveness of ChAdOx1 by dose in elderly populations in countries with high prevalence of the Gamma variant of SARS-CoV-2. Here, we estimate ChAdOx1 effectiveness by dose against the primary endpoint of RT-PCR-confirmed Covid-19, and secondary endpoints of Covid-19 hospitalization and Covid-19-related death, in adults aged ≥60 years during an epidemic with high Gamma variant prevalence in São Paulo state, Brazil using a matched, test-negative case-control study. Starting 28 days after the first dose, effectiveness of a single dose of ChAdOx1 is 33.4% (95% CI, 26.4–39.7) against Covid-19, 55.1% (95% CI, 46.6–62.2) against hospitalization, and 61.8% (95% CI, 48.9–71.4) against death. Starting 14 days after the second dose, effectiveness of the two-dose schedule is 77.9% (95% CI, 69.2–84.2) against Covid-19, 87.6% (95% CI, 78.2–92.9) against hospitalization, and 93.6% (95% CI, 81.9–97.7) against death. Completion of the ChAdOx1 vaccine schedule affords significantly increased protection over a single dose against mild and severe Covid-19 outcomes in elderly individuals during widespread Gamma variant circulation.

## Abstract (web summary)

Here, the authors investigate the effectiveness of the Oxford-AstraZeneca (ChAdOx1) vaccine during extensive Gamma variant SARS-CoV-2 circulation in São Paulo state, Brazil, and find that a two-dose regime is more effective than one dose against mild to severe Covid-19 outcomes in older adults.

## Introduction

Multiple vaccines against severe acute respiratory syndrome coronavirus 2 (SARS-CoV-2), the etiologic agent that causes coronavirus disease 19 (Covid-19), have been developed, proven efficacious, and deployed in mass vaccination campaigns^1–3^. Prominent among these vaccines, particularly in lower-income and middle-income countries, is the viral vector vaccine, ChAdOx1^4^. Randomized controlled trials (RCT) of ChAdOx1 delivered with a four-week inter-dose interval demonstrated 70.4% (95% CI: 54.8–80.6) efficacy against symptomatic Covid-19 in the period starting 14 days after the second vaccine dose^4^, and 64.1% 95% CI: (50.5–73.9) starting at 21 days following the first dose^5^. Based on measured immunogenicity and efficacy following a single dose, many countries have implemented a dose-spacing strategy that uses an inter-dose interval of up to 12 weeks to maximize vaccine coverage^6^ and has been endorsed by the World Health Organization (WHO)^7^.

The emergence of variants of concern (VOC) associated with decreased neutralization activity has created an urgent need to continuously monitor vaccine effectiveness^8^. Recent evidence has suggested reduced effectiveness of a single dose of ChAdOx1 against the Gamma and Delta VOCs^9,10^. Local Gamma VOC circulation has been observed in countries in Latin America which are using ChAdOx1 in mass vaccination^11^. A key question for these countries is the effectiveness of ChAdOx1 by dose against mild and severe Covid-19 outcomes, particularly in priority populations for vaccination such as the elderly.

The Gamma VOC was first detected in the city of Manaus^12^ and has been a driver of Covid-19 resurgence in Brazil and across South America^13^. The Brazilian national immunization program initiated a mass vaccination campaign in January 2021, which administered ChAdOx1 with three-month dose-spacing. In this work, we evaluate vaccine effectiveness following one and two doses during a epidemic with high Gamma variant prevalence in São Paulo, the most populous state in Brazil. We show that the effectiveness of the completed two-dose schedule is higher than effectiveness of a single dose, and demonstrate robust ChAdOx1 vaccine effectiveness against moderate and severe Covid-19 outcomes in this elderly population.

## Results

### Study setting

**Fig. 1:**
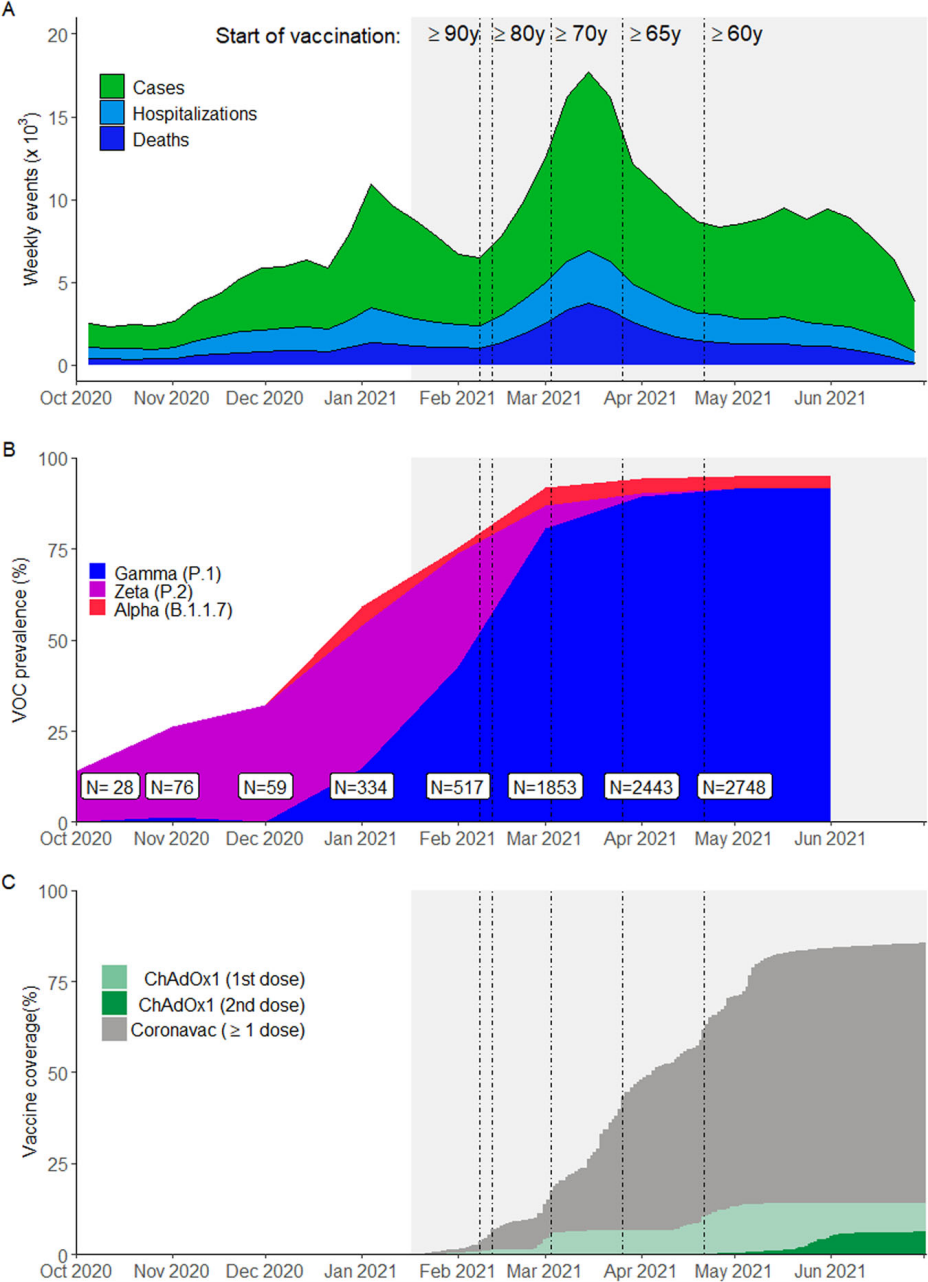
Incidence of reported Covid-19, vaccination coverage, and prevalence of SARS-CoV-2 variants of concern from Oct 1, 2020 to July 2, 2021 in São Paulo State, Brazil. **A** The weekly case count of cases, hospitalizations, and deaths based on positive RT-PCR/Antigen tests for the age group ≥60 years. **B** The monthly prevalence of main SARS-CoV-2 variants of concern among genotyped isolates in the GISAID database^11^ (extraction on July 7 2021). Prevalence was omitted for June and July due to low sample count. **C** Daily cumulative vaccination coverage for age group ≥60 years. Population estimates were obtained from national projections for 2020^35^. Vertical lines, from left to right in each panel, show the dates that adults ≥90, 80–89, 70–79, 65–69, and 60–64 years of age in the general population became eligible for vaccination. The gray shaded area represents the study period. Source data are provided as a Source Data file.

São Paulo State has experienced three Covid-19 epidemic waves, the latest peaking in March 2021, with cumulatively over 3.89 million reported cases, 430,000 hospitalizations, and 130,000 deaths due to Covid-19 as of 9 July 2021^14^ (Fig. 1A). During the second and third waves, the Gamma variant increased in prevalence, reaching 80.2% from March to May 2021 among sequenced isolates, to become the predominant circulating variant in the state (Fig. 1B). The State Secretary of Health of São Paulo (SES-SP) initiated a mass vaccination campaign on 17 January 2021, prioritizing healthcare workers and elderly populations. Two primary vaccines are being distributed: a two-dose regimen of ChAdOx1, separated by a 12-week interval, and a two-dose regimen of CoronaVac, separated by a two- to four-week interval^15^. As of 9 July 2021, 1.61 million doses of ChAdOx1 (1.11 million first doses and 0.51 million second doses) and 9.07 million doses of CoronaVac (5.62 million first doses and 3.45 million second doses) (Fig. 1C) have been administered^16^.

### Study population

**Fig. 2:**
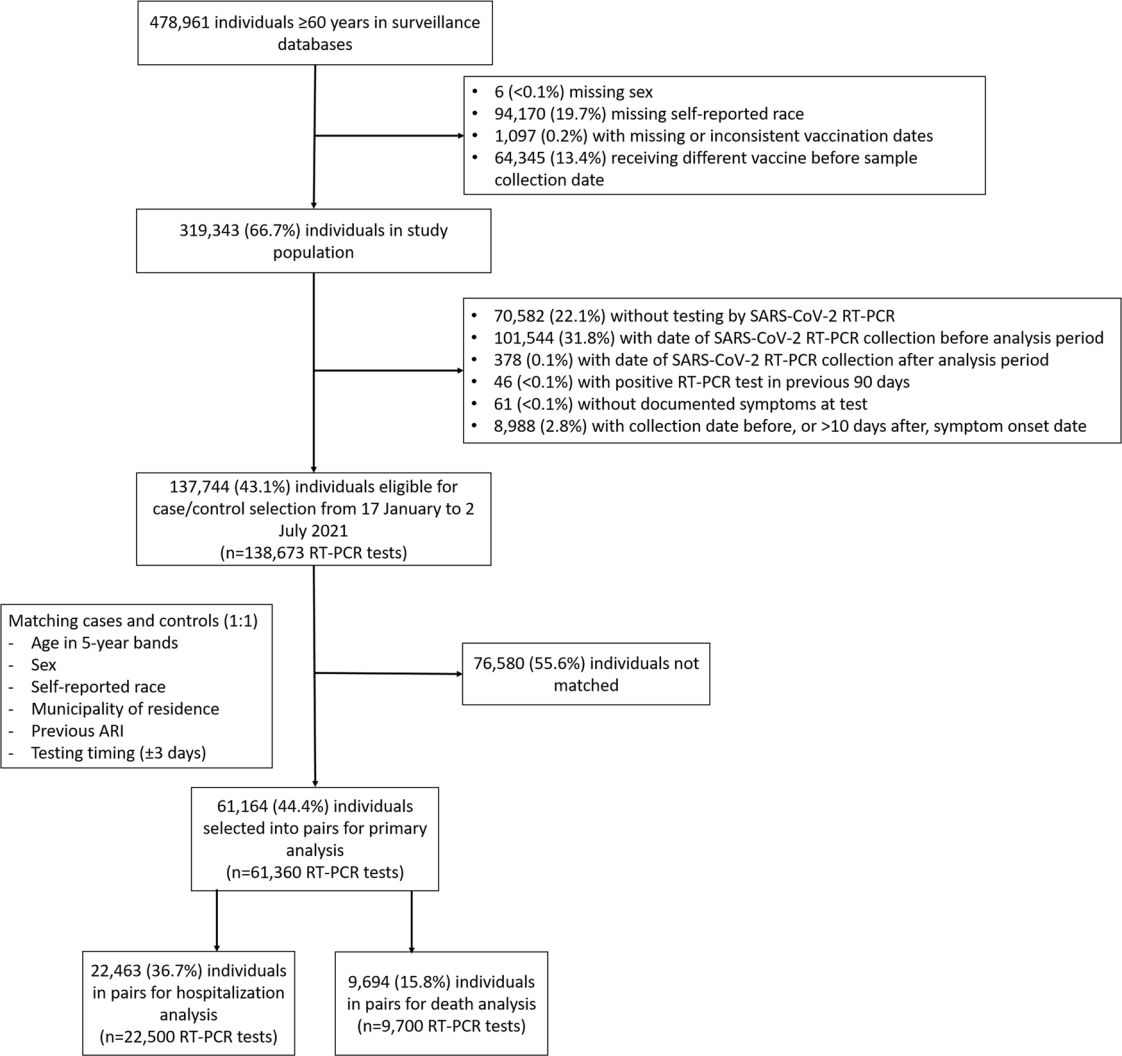
Study flowchart. Flowchart of the identification of the study population from surveillance databases and selection of matched cases and controls.

Among 137,744 individuals eligible for selection as a case or control (Fig. 2), 61,164 (44.4%) who provided 61,360 RT-PCR test results were selected into 30,680 matched case and control pairs. Table 1 and Supplementary Table 1 show the characteristics of eligible individuals and matched cases and controls. Supplementary Tables 2–4 show the distribution of matched pairs according to vaccination status of cases and controls at the time of RT-PCR testing for the analysis of symptomatic Covid-19, hospitalization, and death. Supplementary Fig. 1 shows the timing of discordant pair enrollment, while Supplementary Fig. 2 shows the distribution of intervals between administration of vaccine doses and RT-PCR testing. Among individuals testing positive for SARS-CoV-2 by RT-PCR or rapid antigen tests, 82,061 were eligible for analysis of effectiveness against progression to severe outcomes. Characteristics of this cohort are shown in Supplementary Table 5.

**Table 1:** Characteristics of adults ≥60 years of age who were eligible for matching and selected into case-test negative pairs.

	Eligible cases and controls		Matched pairs	
Characteristics^a^	Test-negative (*n* = 56,676)^b^	Test-positive (*n* = 81,997)^b^	Controls (*n* = 30,680)^b^	Cases (*n* = 30,680)^b^
Demographics				
Age, mean (SD), years	67.90 (7.8)	67.59 (7.2)	66.54 (6.5)	66.55 (6.5)
Male sex, *n* (%)	24,313 (42.9)	39,180 (47.8)	12,976 (42.3)	12,976 (42.3)
Self-reported race, *n* (%)^c^				
White/Branca	40,860 (72.1)	58,565 (71.4)	23,046 (75.1)	23,046 (75.1)
Brown/Pardo	12,484 (22.0)	18,463 (22.5)	6,572 (21.4)	6,572 (21.4)
Black/Preta	2,720 (4.8)	4,063 (5.0)	943 (3.1)	943 (3.1)
Yellow/Amarela	605 (1.1)	890 (1.1)	119 (0.4)	119 (0.4)
Indigenous/Indigena	7 (0.0)	16 (0.0)	–	–
Residence in “Grande São Paulo” Health Region, *n* (%)	39,767 (70.2)	53,540 (65.3)	17,771 (57.9)	17,771 (57.9)
Reported number of comorbidities, *n* (%)^d^				
None	37,434 (66.0)	47,262 (57.6)	20,604 (67.2)	17,520 (57.1)
One or two	18,121 (32.0)	32,093 (39.1)	9507 (31.0)	12,136 (39.6)
Three or more	1121 (2.0)	2642 (3.2)	569 (1.9)	1024 (3.3)
At least one previous ARI event, *n* (%)^e^	2722 (4.8)	1381 (1.7)	299 (1.0)	299 (1.0)
Positive SARS-CoV-2 test result, *n* (%)^f^	310 (0.5)	72 (0.1)	31 (0.1)	19 (0.1)
Vaccination status				
Not vaccinated, *n* (%)	44,285 (78.1)	65,582 (80.0)	24,868 (81.1)	25,215 (82.2)
Single dose, within 0–13 days, *n* (%)	1877 (3.3)	3535 (4.3)	1042 (3.4)	1141 (3.7)
Single dose, 14–27 days, *n* (%)	2543 (4.5)	4406 (5.4)	1427 (4.7)	1380 (4.5)
Single dose, ≥28 days, *n* (%)	6918 (12.2)	7704 (9.4)	3009 (9.8)	2731 (8.9)
2nd dose, within 0–13 days, *n* (%)	303 (0.5)	388 (0.5)	114 (0.4)	107 (0.3)
2nd dose, ≥14 days, *n* (%)	750 (1.3)	382 (0.5)	220 (0.7)	106 (0.3)

^a^Continuous variables are displayed as mean (SD); categorical variables are displayed as *n* (%).

^b^These numbers refer to RT-PCR tests and represent 120,483 individuals for the eligible cases and controls and 53,495 individuals in the matched cases and controls.

^c^Race/skin color as defined by the Brazilian national census bureau (Instituto Nacional de Geografia e Estatísticas)^35^.

^d^Comorbidities included: cardiovascular, renal, neurological, hematological, or hepatic comorbidities, diabetes, chronic respiratory disorder, obesity, or immunosuppression.

^e^Prior to the start of the study on 17 January, 2021 and after systematic surveillance was implemented on 1 February, 2020. Reported illness with Covid-19 associated symptoms in the eSUS and SIVEP-Gripe databases.

^f^Defined as a positive SARS-CoV-2 RT-PCR or antigen detection test result.

### Vaccine effectiveness against symptomatic Covid-19

**Fig. 3:**
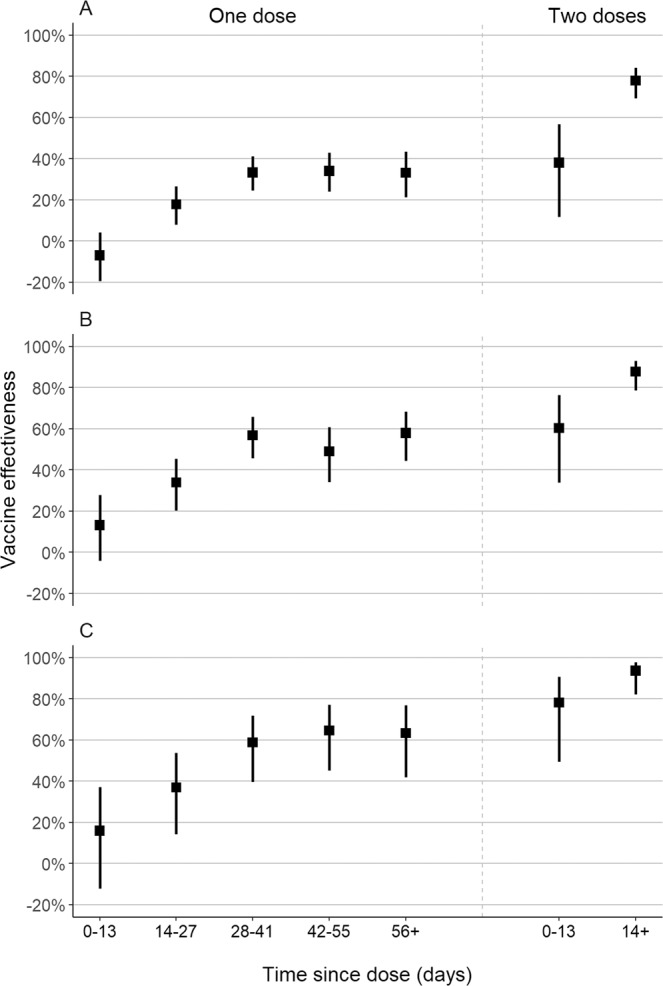
ChAdOx1 vaccine effectiveness by dose. Adjusted vaccine effectiveness (squares) and 95% confidence intervals (lines) of one and two doses of ChAdOx1, by time since vaccination, against symptomatic Covid-19 (**A**), Covid-19 hospitalization (**B**), and Covid-19-related death (**C**) among matched case-control pairs (*n* = 61,360 RT-PCR tests). Source data are provided as a Source Data file.

The adjusted effectiveness of a single dose of ChAdOx1 against symptomatic Covid-19 was 33.4% (95% CI: 26.4–39.7) for the period ≥28 days after administration of the first dose (Table 2). The effectiveness of a single dose reached a plateau after 28 days (Fig. 3), with no increase observed in later time periods. The adjusted effectiveness of the full two-dose schedule against symptomatic Covid-19 was 38.1% (95% CI: 11.9–56.5) in the period 0–13 days after administration of the second dose, and 77.9% (95% CI: 69.2–84.2) in the period ≥14 days after administration of the second dose. The estimated effectiveness in the period 0–13 days following the first dose, which serves as a negative control period to indicate bias, was −7.1% (95% CI: −19.6 to 4.1). Increasing number of comorbidities were significantly associated with increased odds of Covid-19 in the adjusted analyses (aOR 1.54, 95% CI: 1.49–1.60, for one-two comorbidities, and aOR 2.20, 95% CI: 1.98–2.45, for three or more comorbidities compared to no comorbidities). A previous positive SARS-CoV-2 viral test was associated with lower odds of Covid-19 (aOR 0.65, 95% CI: 0.37–1.17). Unadjusted, but matched, analyses provided similar effectiveness estimates (Supplementary Table 6).

**Table 2:** Adjusted effectiveness of a ChAdOx1 against clinical Covid-19 outcomes in adults ≥60 years of age.

	Symptomatic Covid-19 (*n* pairs = 30,680)	Covid-19 hospitalization (*n* pairs = 11,250)	ICU admission (*n* pairs = 4445)	Invasive mechanical ventilation (*n* pairs = 2672)	Covid-19-related death (*n* pairs = 4850)
Vaccine doses and timing	aVE (95% CI)	aVE (95% CI)	aVE (95% CI)	aVE (95% CI)	aVE (95% CI)
Single dose, within 0–13 days vs. unvaccinated*	−7.1% (−19.6–4.1)	13.1% (−4.4–27.7)	−9.1% (−46.6–18.8)	12.7% (−29.2–41)	16.1% (−11.9–37.2)
Single dose, 14–27 days vs. unvaccinated*	17.8% (8.0–26.5)	33.6% (19.9–45.0)	39.6% (15.4–56.8)	51.8% (26.3–68.4)	37.5% (15.2–54.0)
Single dose, ≥28 days vs. unvaccinated*	33.4% (26.4–39.7)	55.1% (46.6–62.2)	50.9% (33.6–63.8)	70.5% (54.9–80.8)	61.8% (48.9–71.4)
Two doses, within 0–13 days vs. unvaccinated*	38.1% (11.9–56.5)	59.2% (32.4–75.4)	50.9% (−41.8–83)	75.2% (−18.7–94.8)	77.8% (49.1–90.3)
Two doses, ≥14 days vs. unvaccinated*	77.9% (69.2–84.2)	87.6% (78.2–92.9)	89.9% (70.9–96.5)	96.5% (81.7–99.3)	93.6% (81.9–97.7)

*aVE* adjusted vaccine effectiveness. *At date of index sample collection for cases and controls.

### Vaccine effectiveness against severe Covid-19 outcomes

In the period starting 28 days after the first dose, the adjusted effectiveness of a single dose was 55.1% (95% CI: 46.6–62.2) against hospitalization, and 61.8% (95% CI: 48.9–71.4) against death (Table 2). The adjusted effectiveness of the two-dose schedule starting 14 days after the second dose was higher: 87.6% (95% CI: 78.2–92.9) against hospitalization, and 93.6% (95% CI: 81.9–97.7) against death (Table 2). Effectiveness against ICU admission and mechanical ventilation was similar to effectiveness against hospitalization (Table 2). In general, vaccine effectiveness in the “bias-indicator” period 0–13 days after the first dose was low. Results were similar when performing different matching schemes (Supplementary Tables 7 and 8), with some small improvements in precision. Analysis of hospitalization, ICU admission, and death among those testing positive for SARS-CoV-2 estimated low effectiveness against progression before 28 days after the first dose, followed by increased effectiveness but with low precision for effectiveness starting 14 days after the second dose (Supplementary Table 9).

### Subgroup analyses

The effectiveness of a single dose against symptomatic Covid-19 was lower among those with reported diabetes (24.2%, 95% CI: 11.0–35.4) than in those without reported diabetes (35.3%, 95% CI: 28.3–41.6) (p_interaction_ = 0.03) (Supplementary Table 10). Similarly, effectiveness was lower among those with at least one reported comorbidity compared to those without a reported comorbidity (Supplementary Table 10). Finally, single-dose effectiveness against hospitalization and death was lower among older individuals, but these analyses lacked sufficient power (Supplementary Table 11).

## Discussion

A key priority for mass vaccination campaigns is to reduce morbidity and mortality in the elderly and other vulnerable populations, especially in the context of limited vaccine supply and VOC emergence. Our test-negative case–control study found that the two-dose schedule of ChAdOx1 in the elderly had robust effectiveness against Covid-19 and severe outcomes during a Covid-19 epidemic with high Gamma variant prevalence in the period starting 14 days after administration of the second dose: 77.9% (95% CI: 69.2–84.2) against symptomatic Covid-19, 87.6% (95% CI: 78.2–92.9) against Covid-19 hospitalization, and 93.6% (95% CI: 81.9–97.7) against Covid-19-related death. However, a single dose of ChAdOx1 in adults 60 years of age had effectiveness of 33.4% (95% CI: 26.4–39.7) against symptomatic Covid-19, 55.1% (95% CI: 46.6–62.2) against hospitalization, and 61.8% (95% CI: 48.9–71.4) against death. Additionally, no clinically significant effectiveness was detected within 28 days of administration of the first dose.

Randomized controlled trials of ChAdOx1 conducted in multiple countries reported pooled vaccine efficacy of 70.4% (95% CI: 54.8–80.6) against symptomatic Covid-19 in the period starting 14 days after the second vaccine dose, and 100% (95% CI, not calculated) against hospitalization for Covid-19^4^. A secondary analysis estimated efficacy of 64.1% (95% CI: 50.5–73.9) against symptomatic Covid-19 starting at 21 days following the first dose^5^. Subsequent observational studies have largely supported the effectiveness of ChAdOx1 against symptomatic Covid-19 and hospitalization in elderly populations^17–20^. In addition, these studies provided further evidence for the effectiveness of a single dose of ChAdOx1 against infection with SARS-CoV-2, symptomatic Covid-19^17^, and hospitalization^18,19^, with onset of clinical effectiveness occurring between 21 and 28 days.

Emerging VOCs have been associated with reduced neutralization by serum from individuals who have been infected with non-VOC strains, and vaccinated^20,21^, including those who are elderly^22^, raising the possibility of decreased effectiveness. An RCT of ChAdOx1 conducted in South Africa found no effectiveness, albeit with low precision, of the two-dose vaccine schedule against mild-to-moderate Covid-19 caused by the Beta VOC^23^. Further evidence from observational studies has suggested reduced vaccine effectiveness against symptomatic disease for a single dose of vaccine against Gamma: 48% (95% CI: 28–63) after 14 days for ChAdOx1^10^, 61% (95% CI: 45–72) after 21 days for mRNA vaccines^24^, and 11% (95% CI: −4 to 23) after 14 days for CoronaVac^25^. However, the complete BNT162b2 schedule has shown robust effectiveness against the Gamma VOC^10^, and a complete schedule of CoronaVac was effective against mild and severe outcomes in settings of high Gamma VOC prevalence^25^. These findings are consistent with reduced effectiveness of a single dose of BNT162b2 and ChAdOx1 against the Delta VOC observed in the UK^9^. Our study adds to this evidence base by estimating single-dose effectiveness of ChAdOx1 over the duration of the inter-dose interval, and demonstrating a substantial increase in effectiveness against Covid-19 and severe outcomes after the second dose in elderly individuals in a setting of high Gamma VOC prevalence.

Our findings have implications for vaccination policy in countries experiencing Covid-19 epidemics with high Gamma variant prevalence. Several countries, including Brazil, are administering the two-dose schedule of ChAdOx1 with a 12-week gap between doses to increase coverage, as WHO currently recommends^7^. The public health benefits of dose-spacing strategies were predicated on robust effectiveness following a single dose^26–28^. In the specific context of VOC emergence and spread, national programs should consider the reduced vaccine effectiveness of a single dose against the Gamma and Delta VOCs in the elderly, together with vaccine supply limitations, speed of vaccination, and logistics, when quantifying the benefits of dose-spacing strategies.

The design of this study lends strength to our findings. The six-month period during which the Gamma variant-associated epidemic and vaccination campaign occurred provided the opportunity to obtain robust estimates of single-dose effectiveness beyond 28 days, and effectiveness of the completed schedule in the same population for direct comparison. The test-negative design reduces bias caused by healthcare-seeking behavior^29^, and we have controlled for additional sources of bias by matching on several predictors of healthcare access and utilization and Covid-19 risk^30^. We used a negative control period of 0–13 days within receiving the first dose to detect bias in our estimates, and found limited measured effectiveness in this period. This null association suggests that matched cases and controls were similar in their propensity to be vaccinated; differences observed for subsequent time periods were likely associated with vaccination rather than underlying characteristics of those who did and did not receive it^31^. The large sample size allowed us to produce robust estimates even against rare outcomes such as death and to perform subgroup analyses.

Our study has several limitations. We could not estimate the effectiveness against Gamma and non-Gamma Covid-19 cases within this study population, as we did not have access to individual-level genetic data on the virus. However, the majority of discordant case-control pairs selected (3728/3834, 97%) received RT-PCR tests after 1 March 2021, after which the prevalence of the Gamma variant among sequenced isolates was 80.2%. In addition, there was likely a proportion of the population that was seropositive without having received a previous positive RT-PCR or rapid antigen test before the study period. These individuals, even if unvaccinated, would be protected from reinfection by natural immunity, thus causing downward bias in our vaccine effectiveness estimates. Controls for the analysis of severe outcomes included controls with mild ARI, who may have had better access to healthcare, leading to bias in our estimates of effectiveness against severe outcomes. Exclusions due to missing data or lack of a matching control affect the generalizability of our results, as individuals who had complete data and were matched might not be representative of the general population. However, different matching schemes including a higher proportion of eligible cases returned similar results. Finally, our results cannot be extrapolated to younger populations.

In a setting of widespread circulation of the SARS-CoV-2 Gamma variant, in the general population of elderly individuals, completion of the two-dose schedule of ChAdOx1 was associated with a significant increase in protection against mild and severe Covid-19 outcomes compared to a single dose.

## Methods

The study was approved by the Ethical Committee for Research of Federal University of Mato Grosso do Sul (CAAE: 43289221.5.0000.0021). We obtained local IRB approval to waive the Free and Informed Consent form. This was possible because the anonymized data bases from the surveillance system were sent to us only after the linkage and did not allow identification of the study participants. The second cohort was selected from within the surveillance data bases described, and did not involve any further prospective follow-up or data collection.

### Study setting

The study setting and design have been described in detail elsewhere^25,32^. We obtained individual-level information on demographic characteristics, comorbidities, SARS-CoV-2 testing, and Covid-19 vaccination by extracting information on 9 July 2021 from the SES-SP laboratory testing registry (GAL), the national surveillance databases for acute respiratory illness (ARI) (e-SUS) and severe ARI (SIVEP-Gripe), and the SES-SP vaccination registry (Vacina Já), containing Covid-19 vaccine information for all individuals vaccinated in São Paulo State (Supplementary Table 12). The surveillance databases cover hospitalizations, as well as primary care, inpatient and specialty outpatient health visits conducted through public and private health systems. Notification of SARS-CoV-2 test results and suspected Covid-19 cases, hospitalizations, and deaths to these systems is compulsory. We retrieved information on SARS-CoV-2 variants from genotyped isolates deposited in the GISAID database^11^. The STROBE checklist is shown in Supplementary Table 13. The protocol, including statistical analysis plan, further details of study design, and power calculations, is publicly available^33^.

### Study population and design

The study population was adults ≥60 years of age who had a residential address in São Paulo State and complete and consistent information between data sources on age, sex, residence, and vaccination and testing status and dates. We selected cases who had an ARI, received a positive SARS-CoV-2 RT-PCR test during the study period of 17 January 2021 to 2 July 2021 with sample collection date within 10 days after symptom onset, and without a positive SARS-CoV-2 RT-PCR test in the previous 90 days. We selected test-negative controls who had an ARI, received a negative SARS-CoV-2 RT-PCR test during the study period with sample collection date within 10 days after symptom onset, and without a positive SARS-CoV-2 RT-PCR test in the previous 90 days or following 14 days. Cases and controls who had received a dose of another Covid-19 vaccine before their RT-PCR test were excluded. We matched one control to each case by date of RT-PCR testing (±3 days), age (in 5-year bands), sex, self-reported race (brown, black, yellow, white, or indigenous)^34^, municipality of residence, and prior ARI (defined as at least one previous symptomatic event that was reported to surveillance systems between 1 February 2020 and 16 January 2021). Each control RT-PCR test could serve as a control for only a single case. To assess the effect of different matching schemes, we performed two sensitivity analyses on the primary results: we matched controls with replacement (so that each control RT-PCR test could be matched to multiple cases), and we matched two controls to each case, with replacement.

To support the analysis of effectiveness against severe outcomes, we additionally estimated effectiveness of the vaccine against progression to severe outcomes among individuals with Covid-19. From the same surveillance database, we selected individuals into a cohort who had an ARI with symptom onset date and sample collection date between 17 January 2021 and 4 June 2021 (28 days before the end of the study period, to allow for reporting of severe outcomes in recently infected individuals), received a positive SARS-CoV-2 RT-PCR or rapid antigen test within 14 days of symptom onset, and were otherwise eligible members of the study population.

### Outcomes and covariates

We estimated the effectiveness of ChAdOx1 against the primary outcome of symptomatic Covid-19 during the period ≥28 days after a single vaccine dose, and 0–13 and ≥14 days after two vaccine doses. Furthermore, we estimated the effectiveness of a single dose during the period 14–27 days after the first dose to understand the onset of protection, and in the period 0–13 days, when the vaccine has no or limited effectiveness^17^. An association during this period may serve as a negative control period to detect unmeasured confounding in the effectiveness estimate in later time periods^31^. In a secondary analysis, we estimated vaccine effectiveness following the first dose in the time windows 28–41 days, 42–55 days, and ≥56 days separately. The reference group for vaccination status was individuals who had not received a first vaccine dose before the date of sample collection.

In addition, we estimated vaccine effectiveness against secondary outcomes of Covid-19 hospitalization, ICU admission with Covid-19, mechanical ventilation for Covid-19, and Covid-19-related death. We estimated single-dose effectiveness during the period ≥28 days after the first dose for all outcomes within subgroups defined by age (60–69 years vs. ≥70 years), sex, number of chronic comorbidities (none vs. at least one), reported cardiovascular disease, reported diabetes (the two most common reported comorbidities), and region of residence (“Grande São Paulo” health region vs. others). For the analysis among test-positive individuals, we analyzed the effectiveness of the vaccine against progression to hospitalization within 21 days of symptom onset, ICU admission within 21 days of symptom onset, and against death within 28 days of symptom onset.

### Statistics

We performed conditional logistic regression to estimate vaccine effectiveness for each time window following vaccination, accounting for the matched design. Multivariable models were adjusted for the number of reported comorbidities (categorized as none, one-two, and at least three), previous positive SARS-CoV-2 RT-PCR or antigen test, and age as a continuous variable because we used 5-year age bands as a matching factor. For each outcome, we selected matched pairs in which cases had the outcome of interest, and fit the model described above to each subset. For severe outcomes, controls therefore represented test-negative patients from ambulatory and hospital settings who received RT-PCR testing. Finally, we conducted a Cox proportional hazards model to estimate effectiveness against progression within test-positive individuals. To account for variation in incidence and hospitalization practices by time and across the state, we stratified the baseline hazard by week of symptom onset and municipality of residence, and additionally adjusted for the matching factors and additional covariates listed above.

Our protocol specified that we would conduct proposed analyses after achieving ≥80% power to identify a vaccine effectiveness of 40% against symptomatic Covid-19 ≥28 days after a single dose of ChAdOx1, and 80% power to identify 50% effectiveness of two doses ≥14 days after the second dose. The power was estimated by fitting conditional logistic regressions on 1000 simulated datasets. After extracting the surveillance databases on July 9 2021 and generating matched case–control pairs, we determined that the power of the study was >99.8% for each analysis and performed the pre-specified analyses. All data processing and analyses were performed in R, version 4.0.2.

### Reporting summary

Further information on research design is available in the Nature Research Reporting Summary linked to this article.

## Supplementary information

Supplementary information

Reporting Summary

## Data Availability

Deidentified analysis data sets generated from the surveillance and vaccine registry databases are available in the Github repository https://github.com/juliocroda/VebraCOVID-19^33^. Source data are provided as a Source Data file. For Fig. 1, vaccine data was obtained from OpenDataSUS (https://opendatasus.saude.gov.br/, access date 2021-07-09) and variant data from GISAID (https://www.gisaid.org/hcov19-variants/, access date 2021-07-07). Source data are provided with this paper.

## Source data

Source Data

## Code availability

Code used to perform power calculations, conduct statistical analysis, and produce figures is available in the repository https://github.com/juliocroda/VebraCOVID-19^33^.
