# Supplementary material for: Effectiveness of ChAdOx1 vaccine in older adults during SARS-CoV-2 Gamma variant circulation in São Paulo
Source: Nat Commun. 2021 Oct 28;12:6220. doi: 10.1038/s41467-021-26459-6 (PMC8553924; doi:10.1038/s41467-021-26459-6)
Supplement: Supplementary file 1 — Supplementary information [file 41467_2021_26459_MOESM1_ESM.docx]

**Supplementary information**

**Supplement to:** Effectiveness of ChAdOx1 vaccine in older adults during SARS-CoV-2 Gamma variant circulation in São Paulo


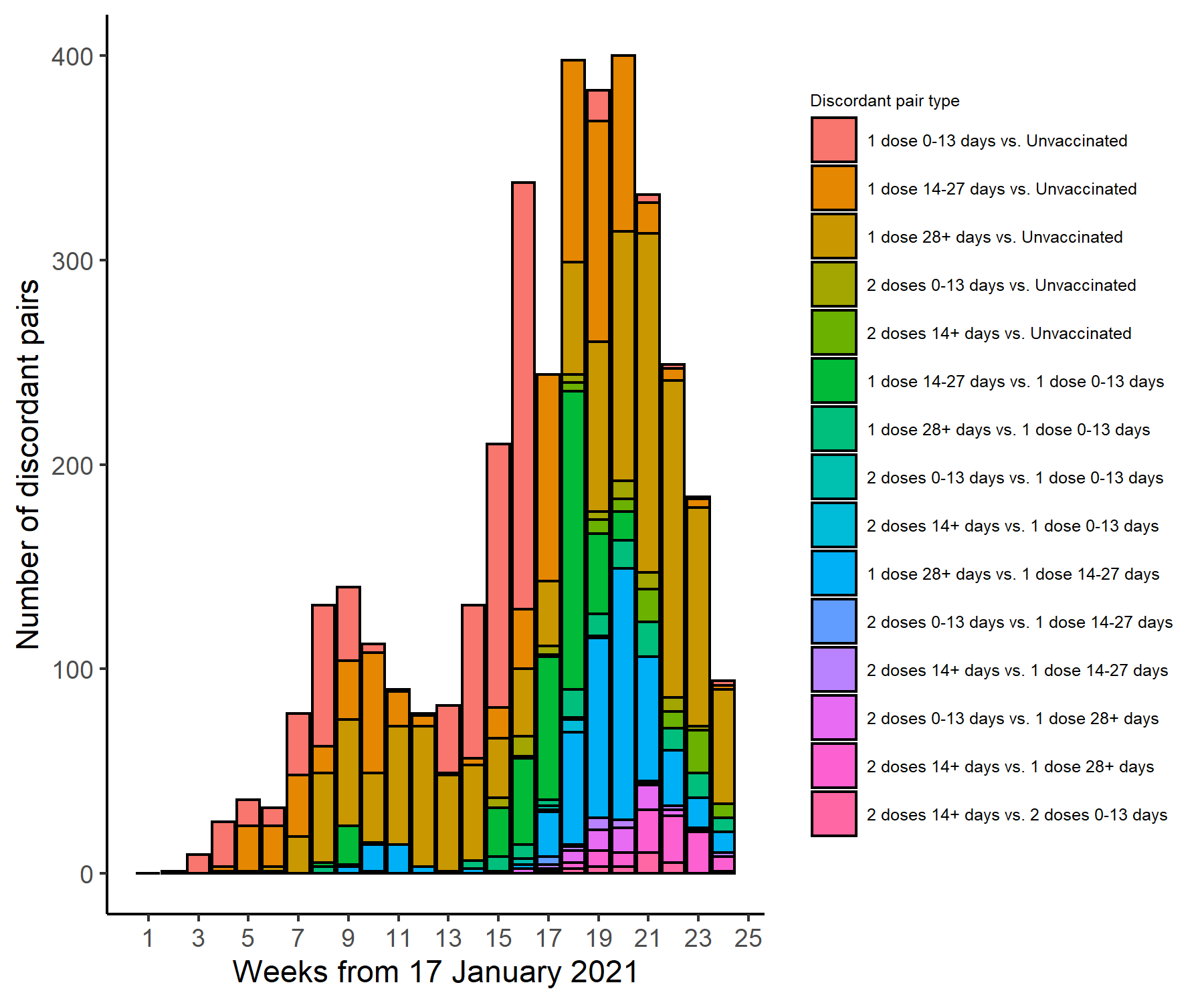
Supplementary Figure 1. **Timing of discordant case-control pair selection.** Timing of enrolment of discordant case-control pairs by vaccination category (n=4,046 discordant pairs). Source data are provided as a Source Data file.

Supplementary Figure 2. **Timing of RT-PCR sample collection date relative to vaccination.** Timing of RT-PCR sample collection date relative to 1^st^ (left column) and 2^nd^ (right column) vaccine dose date, among cases (top row) and controls (bottom row) who were vaccinated during the study period.
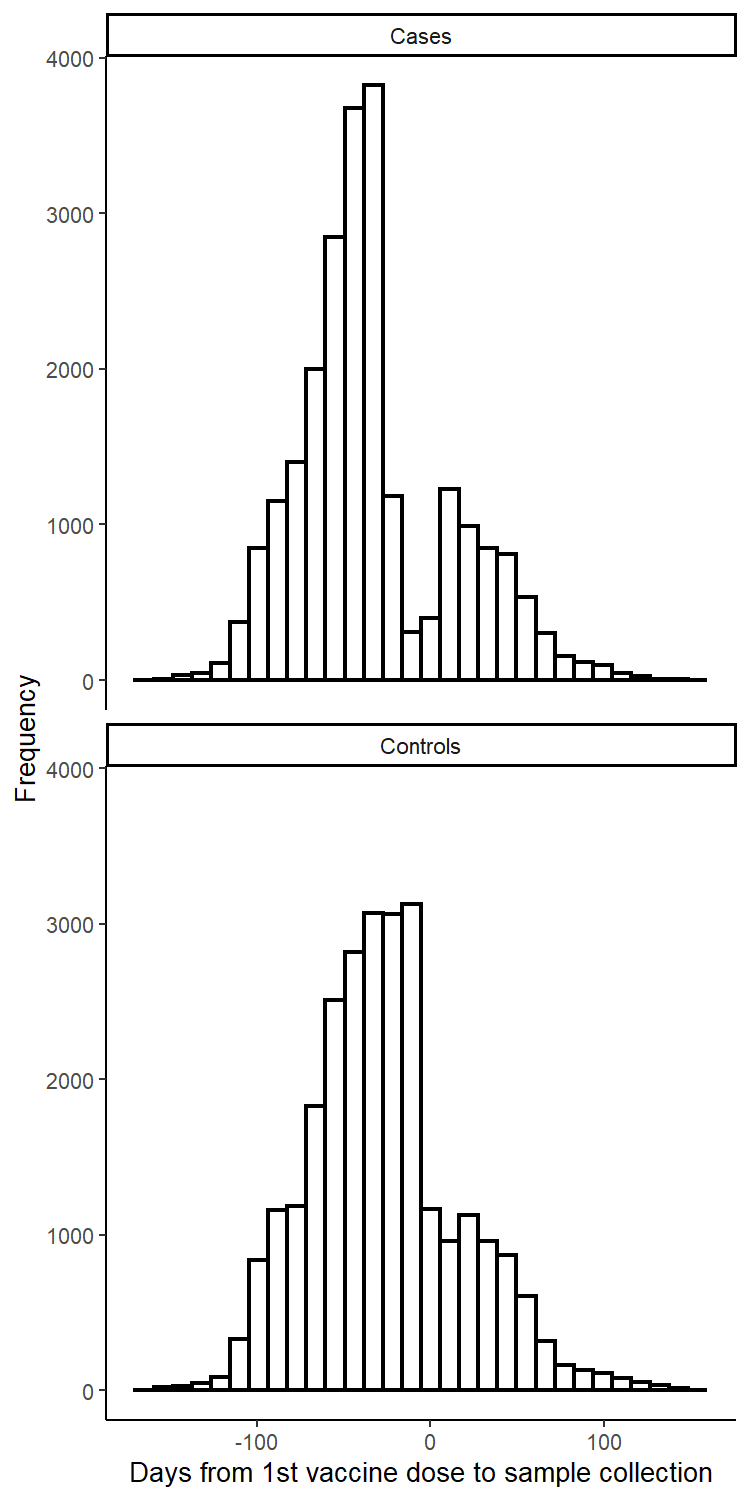

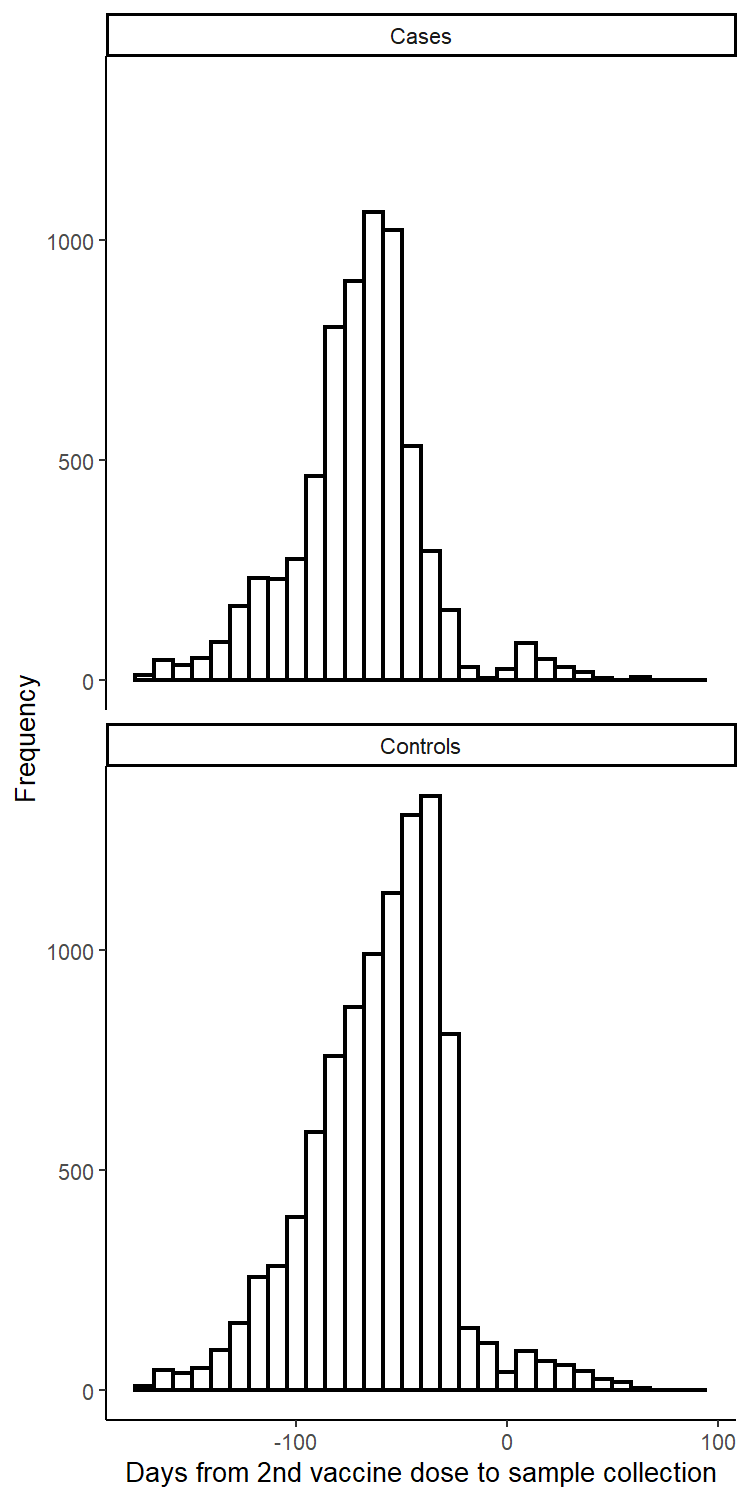
 Source data are provided as a Source Data file.

Supplementary Table 1. Characteristics of adults ≥60 years of age who were eligible for matching and selected into case-test negative pairs.

|  | **Eligible cases and controls** | | **Matched pairs** | |
| --- | --- | --- | --- | --- |
| **Characteristics*** | **Test-negative (n=56,676)^** | **Test-positive (n=81,997)^** | **Controls (n=30,680)^** | **Cases (n=30,680)^** |
| **Demographics** |  |  |  |  |
| Age categories, n (%) |  |  |  |  |
| 60-69 years | 39,842 (70.3) | 58,181 (71.0) | 23,684 (77.2) | 23,684 (77.2) |
| 70-79 years | 10,346 (18.3) | 15,963 (19.5) | 4,858 (15.8) | 4,858 (15.8) |
| 80-89 years | 5,570 (9.8) | 6,949 (8.5) | 1,966 (6.4) | 1,966 (6.4) |
| ≥90 years | 918 (1.6) | 904 (1.1) | 172 (0.6) | 172 (0.6) |
| **Comorbidities** |  |  |  |  |
| Cardiovascular disease, n (%) | 13,069 (23.1) | 24,456 (29.8) | 6,865 (22.4) | 9,429 (30.7) |
| Diabetes, n (%) | 8,078 (14.3) | 16,592 (20.2) | 4,308 (14.0) | 6,319 (20.6) |
| **Clinical outcomes and vaccine timing** |  |  |  |  |
| ARI-related hospitalization, n/n not missing (%) | 7,531/56,468 (13.3) | 30,189/81,515 (37.0) | 3,818/30,581 (12.5) | 11,250/ 30,502 (36.9) |
| ARI-related death, n/n not missing (%) | 2,571/55,495 (4.6) | 14,082/78,921 (17.8) | 1,321/30,144 (4.4) | 4,850/29,543 (16.4) |
| Interval between symptom onset and RT-PCR testing, median (IQR), days | 3 (2-5) | 4 (3-6) | 3 (2-5) | 4 (2-6) |
| Interval between symptom onset and hospitalization, median (IQR), days | 3 (1-5) | 7 (4-10) | 2 (1-5) | 7 (4-10) |
| Interval between symptom onset and death, median (IQR), days | 8 (4-15) | 16 (11-24) | 8.5 (4-16) | 17 (11-25) |
| Interval between 1st and 2nd dose, median (IQR), days | 85 (84-90) | 86 (84-92) | 85 (84-90) | 86 (84-92) |
| Interval between 1st dose and RT-PCR testing, median (IQR), days | 37 (21-57) | 28 (15-48) | 33 (18-50) | 30 (15-48) |
| Interval between 2nd dose and RT-PCR testing, median (IQR), days | 21 (12-33) | 13 (8-24) | 20 (10-34) | 13 (8-24) |

*Continuous variables are displayed as mean (SD); categorical variables are displayed as n (%).

^These numbers refer to RT-PCR tests and represent 120,483 individuals for the eligible cases and controls and 53,495 individuals in the matched cases and controls.

# Supplementary Table 2. Distribution of concordant and discordant matched case-control pairs.

|  | Case unvaccinated | Case 1 dose,  0-13 days | Case 1 dose,  14-27 days | Case 1 dose, ≥28 days | Case 2 doses,  0-13 days | Case 2 doses, ≥14 days |
| --- | --- | --- | --- | --- | --- | --- |
| Control unvaccinated | 23,575 | 471 | 313 | 475 | 23 | 11 |
| Control 1 dose,  0-13 days | 463 | 381 | 155 | 41 | 2 | 0 |
| Control 1 dose,  14-27 days | 353 | 205 | 658 | 206 | 1 | 4 |
| Control 1 dose,  ≥28 days | 734 | 71 | 232 | 1922 | 25 | 25 |
| Control 2 doses,  0-13 days | 30 | 6 | 6 | 22 | 41 | 9 |
| Control 2 doses,  ≥14 days | 60 | 7 | 16 | 65 | 15 | 57 |

# Supplementary Table 3. Distribution of concordant and discordant matched case-control pairs with hospitalized cases.

|  | Case unvaccinated | Case 1 dose,  0-13 days | Case 1 dose,  14-27 days | Case 1 dose, ≥28 days | Case 2 doses,  0-13 days | Case 2 doses, ≥14 days |
| --- | --- | --- | --- | --- | --- | --- |
| Control unvaccinated | 8,454 | 190 | 138 | 163 | 11 | 5 |
| Control 1 dose,  0-13 days | 222 | 143 | 62 | 17 | 0 | 0 |
| Control 1 dose,  14-27 days | 177 | 79 | 221 | 68 | 0 | 0 |
| Control 1 dose,  ≥28 days | 369 | 25 | 77 | 632 | 17 | 10 |
| Control 2 doses,  0-13 days | 20 | 4 | 5 | 11 | 21 | 4 |
| Control 2 doses,  ≥14 days | 36 | 2 | 8 | 22 | 8 | 29 |

# Supplementary Table 4. Distribution of concordant and discordant matched case-control pairs with cases who died.

|  | Case unvaccinated | Case 1 dose,  0-13 days | Case 1 dose,  14-27 days | Case 1 dose, ≥28 days | Case 2 doses,  0-13 days | Case 2 doses, ≥14 days |
| --- | --- | --- | --- | --- | --- | --- |
| Control unvaccinated | 3,734 | 77 | 54 | 54 | 4 | 2 |
| Control 1 dose,  0-13 days | 103 | 55 | 16 | 7 | 0 | 0 |
| Control 1 dose,  14-27 days | 75 | 30 | 90 | 25 | 0 | 0 |
| Control 1 dose,  ≥28 days | 158 | 12 | 30 | 240 | 5 | 2 |
| Control 2 doses,  0-13 days | 9 | 3 | 4 | 6 | 9 | 1 |
| Control 2 doses,  ≥14 days | 13 | 2 | 3 | 12 | 4 | 11 |

| **Characteristics*** | **COVID-19 with positive RT-PCR or rapid antigen test (n=82,061)** |
| --- | --- |
| Age, mean (SD), years | 67.7 (7.3) |
| Male sex, n (%) | 39,372 (48.0) |
| Self-reported race^†^: White/Branca, n(%) | 58,357 (71.1) |
| Self-reported race^†^: Brown/Pardo, n(%) | 18,649 (22.7) |
| Self-reported race^†^: Black/Preta, n(%) | 4,120 (5.0) |
| Self-reported race^†^: Yellow/ Amarela, n(%) | 919 (1.1) |
| Self-reported race^†^: Indigenous/Indigena, n(%) | 16 (0.0) |
| Reported number of comorbidities^‡^, n (%) |  |
| None | 46,500 (56.7) |
| One or two | 32,818 (40.0) |
| Three or more | 2,743 (3.3) |
| Not vaccinated, n (%) | 69,271 (84.4) |
| Single dose, within 0-13 days, n (%) | 3,643 (4.4) |
| Single dose, 14-27 days, n (%) | 4,518 (5.5) |
| Single dose, ≥28 days, n (%) | 4,305 (5.2) |
| 2nd dose, within 0-13 days, n (%) | 255 (0.3) |
| 2nd dose, ≥14 days, n (%) | 69 (0.1) |
| Hospitalization, n (%) | 33,034 (40.3%) |
| Death, n (%) | 15,611 (19.0%) |

*Continuous variables are displayed as mean (SD); categorical variables are displayed as n (%).

^These numbers refer to RT-PCR tests and represent 120,483 individuals for the eligible cases and controls and 53,495 individuals in the matched cases and controls.

^†^Race/skin color as defined by the Brazilian national census bureau (Instituto Nacional de Geografia e Estatísticas).^35^

^‡^Comorbidities included: cardiovascular, renal, neurological, hematological, or hepatic comorbidities, diabetes, chronic respiratory disorder, obesity, or immunosuppression.

Supplementary Table 5. Characteristics of adults ≥60 years of age who were eligible for inclusion in the cohort of individuals with RT-PCR- or antigen-confirmed COVID-19.

Supplementary Table 6. Unadjusted effectiveness of a ChAdOx1 against clinical Covid-19 outcomes in adults ≥60 years of age

|  | **Symptomatic Covid-19**  **(n pairs=48,452)** | **Covid-19 hospitalization**  **(n pairs=18,552)** | **ICU admission**  **(n pairs=7,444)** | **Invasive mechanical ventilation**  **(n pairs=4,391)** | **Covid-19-related death**  **(n pairs=8,109)** |
| --- | --- | --- | --- | --- | --- |
| **Vaccine doses and timing** | **VE (95% CI)** | **VE (95% CI)** | **VE (95% CI)** | **VE (95% CI)** | **VE (95% CI)** |
| Single dose, within 0-13 days vs. unvaccinated | -5.1% (-17.2-5.8) | 13.7% (-2.0-26.9) | 4.2% (-24.4-26.3) | 18.7% (-14.0-42.1) | 19.0% (-4.9-37.5) |
| Single dose, 14-27 days vs. unvaccinated | 17.6% (8.0-26.3) | 29.5% (16.4-40.6) | 45.0% (26.3-59.0) | 50.9% (28.8-66.1) | 40.1% (20.9-54.6) |
| Single dose, ≥28 days vs. unvaccinated | 34.1% (27.3-40.3) | 52.9% (44.9-59.8) | 54.5% (40.8-65.0) | 71.1% (58.4-80) | 62.0% (50.8-70.6) |
| Two doses, within 0-13 days vs. unvaccinated | 39.0% (13.4-57) | 53.5% (25.6-71) | 41.8% (-38.9-75.6) | 72.4% (3.2-92.1) | 73.9% (44.2-87.8) |
| Two doses, ≥14 days vs. unvaccinated | 76.8% (67.8-83.3) | 83.6% (72.4-90.2) | 87.3% (66.4-95.2) | 95.6% (79.7-99.0) | 92.5% (80.0-97.2) |

Supplementary Table 7. Adjusted effectiveness of a ChAdOx1 against clinical Covid-19 outcomes in adults ≥60 years of age, when controls were sampled with replacement.

|  | **Symptomatic Covid-19**  **(n pairs=48,452)** | **Covid-19 hospitalization**  **(n pairs=18,552)** | **ICU admission**  **(n pairs=7,444)** | **Invasive mechanical ventilation**  **(n pairs=4,391)** | **Covid-19-related death**  **(n pairs=8,109)** |
| --- | --- | --- | --- | --- | --- |
| **Vaccine doses and timing** | **aVE (95% CI)** | **aVE (95% CI)** | **aVE (95% CI)** | **aVE (95% CI)** | **aVE (95% CI)** |
| Single dose, within 0-13 days vs. unvaccinated | -4.8% (-14.1-3.7) | 0.7% (-14.0-13.5) | -20.6% (-50.9-3.7) | 8.9% (-22.4-32.2) | 9.3% (-12.1-26.7) |
| Single dose, 14-27 days vs. unvaccinated | 13.2% (5.3-20.4) | 27.3% (16.0-37.1) | 33.4% (15.0-47.8) | 46.7% (26.0-61.7) | 35% (18.1-48.4) |
| Single dose, ≥28 days vs. unvaccinated | 34.4% (28.7-39.6) | 56.9% (50.2-62.7) | 54.9% (42.1-64.8) | 69.8% (57.5-78.6) | 62% (51.8-70.1) |
| Two doses, within 0-13 days vs. unvaccinated | 28.4% (5.3-45.8) | 47.4% (22.3-64.3) | 42.4% (-17.1-71.6) | 76.4% (26.7-92.4) | 69.5% (42.8-83.7) |
| Two doses, ≥14 days vs. unvaccinated | 74.4% (66.2-80.6) | 87.0% (79.4-91.8) | 89.7% (76.1-95.6) | 96.9% (87.8-99.2) | 92.2% (82.2-96.6) |

aVE: Adjusted vaccine effectiveness. In all adjusted models, conditional logistic regression included age as a continuous variable, number of reported comorbidities (none vs. one-two vs. three or more), and prior positive SARS-CoV-2 PCR or rapid antigen test.

Supplementary Table 8. Adjusted effectiveness of a ChAdOx1 against clinical Covid-19 outcomes in adults ≥60 years of age, when two controls per case were sampled with replacement.

|  | **Symptomatic Covid-19**  **(n pairs=48,452)** | **Covid-19 hospitalization**  **(n pairs=18,552)** | **ICU admission**  **(n pairs=7,444)** | **Invasive mechanical ventilation**  **(n pairs=4,391)** | **Covid-19-related death**  **(n pairs=8,109)** |
| --- | --- | --- | --- | --- | --- |
| **Vaccine doses and timing** | **aVE (95% CI)** | **aVE (95% CI)** | **aVE (95% CI)** | **aVE (95% CI)** | **aVE (95% CI)** |
| Single dose, within 0-13 days vs. unvaccinated | -5.9% (-15.3-2.8) | -2.1% (-16.7-10.7) | -14.7% (-41.7-7.1) | 14.4% (-14.0-35.8) | 15% (-5.2-31.4) |
| Single dose, 14-27 days vs. unvaccinated | 15.5% (7.7-22.6) | 34.4% (24.3-43.1) | 41.3% (25.5-53.8) | 50.0% (31.1-63.8) | 48.6% (35.0-59.4) |
| Single dose, ≥28 days vs. unvaccinated | 36.1% (30.6-41.2) | 58.4% (52.1-63.8) | 58.5% (47.7-67.1) | 66.3% (54-75.4) | 62.5% (52.7-70.3) |
| Two doses, within 0-13 days vs. unvaccinated | 28.6% (4.2-46.8) | 58.4% (38.6-71.8) | 52.6% (7-75.9) | 49.0% (-31.5-80.2) | 71.8% (47.2-85) |
| Two doses, ≥14 days vs. unvaccinated | 74.6% (65.7-81.2) | 85.4% (77.2-90.7) | 83.5% (65.2-92.2) | 93.5% (78.4-98.1) | 87.9% (73.7-94.4) |

aVE: Adjusted vaccine effectiveness. In all adjusted models, conditional logistic regression included age as a continuous variable, number of reported comorbidities (none vs. one-two vs. three or more), and prior positive SARS-CoV-2 PCR or rapid antigen test.

Supplementary Table 9. Adjusted one- and two-dose effectiveness against progression to Covid-19 hospitalization, Covid-19 ICU admission, and Covid-19-related death among individuals testing positive for SARS-CoV-2 (n=102,842)

|  | **Covid-19 hospitalization within 21 days of symptom onset** | **Covid-19 ICU admission within 21 days of symptom onset** | **Covid-19 death within 28 days of symptom onset** |
| --- | --- | --- | --- |
| **Vaccine doses and timing** | **aVE (95% CI)** | **aVE (95% CI)** | **aVE (95% CI)** |
| Single dose, within 0-13 days vs. unvaccinated | 1.9% (-3.1-6.6) | 6.0% (-2.3-13.5) | 14.9% (7.8-21.4) |
| Single dose, 14-27 days vs. unvaccinated | 27.7% (23.0-32) | 28.6% (20.4-36.0) | 35.2% (28.5-41.2) |
| Single dose, ≥28 days vs. unvaccinated | 36.7% (32.7-40.4) | 36.2% (29.2-42.5) | 43.0% (37.6-48.0) |
| Two doses, within 0-13 days vs. unvaccinated | 36.9% (22.9-48.4) | 40.2% (15.7-57.6) | 45.1% (26.2-59.2) |
| Two doses, ≥14 days vs. unvaccinated | 34.8% (-4.0-59.1) | 46.3% (-28.1-77.5) | 19.3% (-46.4-55.5) |

aVE: Adjusted vaccine effectiveness. In all adjusted models, stratified Cox proportional hazards regression included age (restricted cubic spline), sex, self-reported race, number of reported comorbidities (none vs. one-two vs. three or more), and prior positive SARS-CoV-2 PCR or rapid antigen test. Baseline hazard was stratified by municipality of residence and week of symptom onset.

Supplementary Table 10. Adjusted single-dose vaccine effectiveness against symptomatic Covid-19, ≥28 days after first dose, within subgroups

|  | aVE* (95% CI) | p-value for interaction^†^ |
| --- | --- | --- |
| **Age** |  |  |
| Age 60-69 years | 34.5% (26.6-41.5) | 0.83 |
| Age ≥70 years | 29.7% (13.3-42.9) |  |
| **Sex** |  |  |
| Females | 31.3% (21.6-39.9) | 0.39 |
| Males | 36.0% (25.6-44.9) |  |
| **Comorbidities** |  |  |
| None | 37.0% (29.6-43.5) | 0.02 |
| One or more | 27.0% (17.2-35.6) |  |
| **CVD** |  |  |
| Not reported | 34.9% (27.7-41.5) | 0.16 |
| Reported | 28.7% (18.0-38.0) |  |
| **Diabetes mellitus** |  |  |
| Not reported | 35.3% (28.3-41.6) | 0.03 |
| Reported | 24.2% (11.0-35.4) |  |
| **Health regional area** |  |  |
| “Grande São Paulo” | 28.3% (16.3-38.5) | 0.50 |
| Not “Grande São Paulo” | 37.2% (28.4-44.9) |  |

* All models adjusted for age as a continuous variable, number of reported comorbidities (none vs. one-two vs. three or more) and previous positive SARS-CoV-2 RT-PCR test

^†^ p-value from a two-sided likelihood ratio test comparing the primary model with a nested model with an interaction between each variable of interest and vaccination with a single dose ≥28 days before RT-PCR sample collection date.

Supplementary Table 11. Adjusted one- and two-dose effectiveness against Covid-19 hospitalization and Covid-19-related death by age

|  | **Adjusted one-dose VE* (95% CI)** | **p-value for interaction**^†^ | **Adjusted two-dose VE* (95% CI)** | **p-value for interaction**^‡^ |
| --- | --- | --- | --- | --- |
| **Covid-19 hospitalization** |  | | | |
| Age 60-69 years | 60.7% (51.1-68.4) | 0.09 | 100.0% (N/A) | <0.001 |
| Age ≥70 years | 41.9% (22.5-56.5) |  | 80.8% (65.2-89.4) |  |
| **Covid-19-related death** |  | | | |
| Age 60-69 years | 72.1% (57.6-81.7) | 0.12 | 100.0% (N/A) | 0.18 |
| Age ≥70 years | 48.0% (22.1-65.3) |  | 89.9% (69.7-96.7) |  |

* All models adjusted for age as a continuous variable, number of reported comorbidities (none vs. one-two vs. three or more) and previous positive SARS-CoV-2 RT-PCR test

^†^ p-value from a two-sided likelihood ratio test comparing the primary model with a nested model with an interaction between each variable of interest and vaccination with a single dose ≥28 days before RT-PCR sample collection date.

^‡^ p-value from a two-sided likelihood ratio test comparing the primary model with a nested model with an interaction between each variable of interest and vaccination with two doses ≥14 days before RT-PCR sample collection date.

| **Database acronym** | **Domain** | **Comment** |
| --- | --- | --- |
| “Vacina Já” | State COVID-19 vaccination registry | State vaccination system, transferred daily to the National Immunization System (SIPNI) |
| SIVEP-Gripe (“Sistema de Informação da Vigilância Epidemiológica da Gripe”) | National surveillance database of severe acute respiratory illnesses | Contains all suspected or confirmed COVID-19 hospitalizations and deaths. In São Paulo State, there is a protocol for *post mortem* RT-PCR SARS-CoV-2 testing for suspected COVID-19 deaths that are also notified to SIVEP-Gripe. |
| e-SUS | National surveillance system of suspected cases of COVID-19 from mild to moderate "influenza like illness" | Contains notifications from primary health care and specialty outpatient visits. |
| GAL (“Gerenciador de Ambiente Laboratorial”) | State laboratory testing registry of the network of public health laboratories | In São Paulo State, diagnostic tests performed in GAL are automatically merged into eSUS and SIVEP-Gripe |

Supplementary Table 12. Description of data sources.

Supplementary Table 13. STROBE Statement—Checklist of items that should be included in reports of case-control studies

|  | Item No | Recommendation | Page No |
| --- | --- | --- | --- |
| **Title and abstract** | 1 | (*a*) Indicate the study’s design with a commonly used term in the title or the abstract | 2 |
|  |  | (*b*) Provide in the abstract an informative and balanced summary of what was done and what was found | 2 |
| Introduction | | | |
| Background/rationale | 2 | Explain the scientific background and rationale for the investigation being reported | 3 |
| Objectives | 3 | State specific objectives, including any prespecified hypotheses | 3 |
| Methods | | | |
| Study design | 4 | Present key elements of study design early in the paper | 9-10 |
| Setting | 5 | Describe the setting, locations, and relevant dates, including periods of recruitment, exposure, follow-up, and data collection | 9-10 |
| Participants | 6 | (*a*) Give the eligibility criteria, and the sources and methods of case ascertainment and control selection. Give the rationale for the choice of cases and controls | 10 |
|  |  | (*b*) For matched studies, give matching criteria and the number of controls per case | 10 |
| Variables | 7 | Clearly define all outcomes, exposures, predictors, potential confounders, and effect modifiers. Give diagnostic criteria, if applicable | 10-11;  protocol |
| Data sources/ measurement | 8* | For each variable of interest, give sources of data and details of methods of assessment (measurement). Describe comparability of assessment methods if there is more than one group | 9 |
| Bias | 9 | Describe any efforts to address potential sources of bias | 9-11 |
| Study size | 10 | Explain how the study size was arrived at | 4; Fig2 |
| Quantitative variables | 11 | Explain how quantitative variables were handled in the analyses. If applicable, describe which groupings were chosen and why | 10-11; Protocol |
| Statistical methods | 12 | (*a*) Describe all statistical methods, including those used to control for confounding | 10-11 |
|  |  | (*b*) Describe any methods used to examine subgroups and interactions | 11 |
|  |  | (*c*) Explain how missing data were addressed | Protocol |
|  |  | (*d*) If applicable, explain how matching of cases and controls was addressed | 11 |
|  |  | (*e*) Describe any sensitivity analyses | 11 |
| Results | | | |
| Participants | 13* | (a) Report numbers of individuals at each stage of study—eg numbers potentially eligible, examined for eligibility, confirmed eligible, included in the study, completing follow-up, and analysed | 4; Fig 2, Supp Mat |
|  |  | (b) Give reasons for non-participation at each stage | Fig 2 |
|  |  | (c) Consider use of a flow diagram | Fig 2 |
| Descriptive data | 14* | (a) Give characteristics of study participants (eg demographic, clinical, social) and information on exposures and potential confounders | 4; Table 1 |
|  |  | (b) Indicate number of participants with missing data for each variable of interest | Table 1 |
| Outcome data | 15* | Report numbers in each exposure category, or summary measures of exposure | Table 1; Supp Mat |
