# Supplementary material for: Effectiveness of ChAdOx1 vaccine in older adults during SARS-CoV-2 Gamma variant circulation in São Paulo
Source: Nat Commun. 2021 Oct 28;12:6220. doi: 10.1038/s41467-021-26459-6 (PMC8553924; doi:10.1038/s41467-021-26459-6)
Supplement: Supplementary file 2 — Reporting Summary [file 41467_2021_26459_MOESM2_ESM.pdf]

nature portfolio

Corresponding author(s): Julio Croda

Last updated by author(s): Sep 28, 2021

Reporting Summary

Nature Portfolio wishes to improve the reproducibility of the work that we publish. This form provides structure for consistency and transparency in reporting. For further information on Nature Portfolio policies, see our Editorial Policies and the Editorial Policy Checklist.

Statistics

For all statistical analyses, confirm that the following items are present in the figure legend, table legend, main text, or Methods section.

n/a	Confirmed	
☐	☒	The exact sample size (*n*) for each experimental group/condition, given as a discrete number and unit of measurement
☐	☒	A statement on whether measurements were taken from distinct samples or whether the same sample was measured repeatedly
☐	☒	The statistical test(s) used AND whether they are one- or two-sided<br>*Only common tests should be described solely by name; describe more complex techniques in the Methods section.*
☐	☒	A description of all covariates tested
☒	☐	A description of any assumptions or corrections, such as tests of normality and adjustment for multiple comparisons
☐	☒	A full description of the statistical parameters including central tendency (e.g. means) or other basic estimates (e.g. regression coefficient) AND variation (e.g. standard deviation) or associated estimates of uncertainty (e.g. confidence intervals)
☐	☒	For null hypothesis testing, the test statistic (e.g. *F*, *t*, *r*) with confidence intervals, effect sizes, degrees of freedom and *P* value noted<br>*Give P values as exact values whenever suitable.*
☒	☐	For Bayesian analysis, information on the choice of priors and Markov chain Monte Carlo settings
☒	☐	For hierarchical and complex designs, identification of the appropriate level for tests and full reporting of outcomes
☐	☒	Estimates of effect sizes (e.g. Cohen's *d*, Pearson's *r*), indicating how they were calculated

Our web collection on statistics for biologists contains articles on many of the points above.

Software and code

Policy information about availability of computer code

Data collection	We used R version 4.0.2 to perform all data collection and processing. Code used to perform power calculations, conduct statistical analysis, and produce figures is available in the repository https://github.com/mhitchings/VEBRA_COVID-19.
Data analysis	We used R version 4.0.2 to perform all data analyses. Code used to perform power calculations, conduct statistical analysis, and produce figures is available in the repository https://github.com/mhitchings/VEBRA_COVID-19.

For manuscripts utilizing custom algorithms or software that are central to the research but not yet described in published literature, software must be made available to editors and reviewers. We strongly encourage code deposition in a community repository (e.g. GitHub). See the Nature Portfolio guidelines for submitting code & software for further information.

Data

Policy information about availability of data

All manuscripts must include a data availability statement. This statement should provide the following information, where applicable:

- Accession codes, unique identifiers, or web links for publicly available datasets
- A description of any restrictions on data availability
- For clinical datasets or third party data, please ensure that the statement adheres to our policy

Deidentified analysis data sets generated from the surveillance and vaccine registry databases are available in the repository https://github.com/juliocroda/VebraCOVID-19. Source data are provided as a Source Data file. For Figure 1, vaccine data was obtained from OpenDataSUS (https://opendatasus.saude.gov.br/, access date 2021-07-09) and variant data from GISAID (https://www.gisaid.org/hcov19-variants/, access date 2021-07-07).

Field-specific reporting

Please select the one below that is the best fit for your research. If you are not sure, read the appropriate sections before making your selection.

☒ Life sciences ☐ Behavioural & social sciences ☐ Ecological, evolutionary & environmental sciences

For a reference copy of the document with all sections, see nature.com/documents/nr-reporting-summary-flat.pdf

Life sciences study design

All studies must disclose on these points even when the disclosure is negative.

Sample size	After creating the analysis data set, we estimated power by assuming a given vaccine effectiveness following one and two doses, simulating vaccine assignment assuming such effectiveness, and estimating the power. Code to perform power calculation is provided, as detailed in the code availability statement.
Data exclusions	We describe exclusion criteria in the methods, and detail numbers excluded in the flowchart in Figure 2. Subjects were excluded if they didn't receive an RT-PCR test for SARS-CoV-2, if they were aged <60 years, or had incomplete or inconsistent data on age, sex, residence, or vaccination status between data bases. In addition, we excluded subjects who were vaccinated with CoronaVac or Pfizer COVID-19 vaccines before the date of their RT-PCR test. Finally, some subjects were excluded due to not being matched to a case or control. In total, 61,164 individuals were included in the analysis data set out of 478,961 individuals aged at least 60 years appearing in the surveillance data bases
Replication	We performed two sensitivity analyses with different matching schemes, which produced similar findings to the primary analysis.
Randomization	This is an observational study, so no randomization occurred. We used a matched test-negative study to reduce bias, and adjusted in the study analysis using conditional logistic regression with stratification.
Blinding	This was a retrospective study, so blinding was not done

Reporting for specific materials, systems and methods

We require information from authors about some types of materials, experimental systems and methods used in many studies. Here, indicate whether each material, system or method listed is relevant to your study. If you are not sure if a list item applies to your research, read the appropriate section before selecting a response.

Materials & experimental systems

n/a	Involved in the study
☒	☐ Antibodies
☒	☐ Eukaryotic cell lines
☒	☐ Palaeontology and archaeology
☒	☐ Animals and other organisms
☐	☒ Human research participants
☒	☐ Clinical data
☒	☐ Dual use research of concern

Methods

n/a	Involved in the study
☒	☐ ChIP-seq
☒	☐ Flow cytometry
☒	☐ MRI-based neuroimaging

Human research participants

Policy information about studies involving human research participants

Population characteristics	The average age of participants included in analysis was 66 years, with 42.3% being male. 75% of individuals self-reported white race, and 42.9% had at least one reported comorbidity.
Recruitment	Participants eligible to be selected into the study population were residents of Sao Paulo State, aged >=60 years old, who received an RT-PCR test between 17 January and 2 July 2021. Eligible participants were identified through national surveillance databases. Cases and controls were selected at random from the eligible population, so no self-selection bias was present. Some bias could be introduced by the matching criteria, so we conducted two sensitivity analyses to explore this bias.
Ethics oversight	The study was approved by the Ethical Committee for Research of Federal University of Mato Grosso do Sul (CAAE: 43289221.5.0000.0021).

Note that full information on the approval of the study protocol must also be provided in the manuscript.
